# Effect of Benzyl Alcohol on Main Defense System Components of *Galleria mellonella* (Lepidoptera)

**DOI:** 10.3390/ijms252011209

**Published:** 2024-10-18

**Authors:** Michalina Kazek, Agata Kaczmarek, Anna K. Wrońska, Mieczysława I. Boguś

**Affiliations:** 1 Department of Microbiology, Molecular Genetics and Genomics, Centre of Advanced Materials and Technology CEZAMAT, Warsaw University of Technology, ul. Poleczki 19, 02-822 Warszawa, Poland; michalina.kazek@pw.edu.pl; 2 Museum and Institute of Zoology, Polish Academy of Sciences, ul. Twarda 51/55, 00-818 Warszawa, Poland; akaczmarek@miiz.waw.pl (A.K.); awronska@miiz.waw.pl (A.K.W.); 3 BIOMIBO, ul. Strzygłowska 15, 04-872 Warszawa, Poland

**Keywords:** hemocytes, insect immune system, oxidative stress, HSP, free fatty acid, *Galleria mellonella*

## Abstract

Benzyl alcohol (E1519) is an aromatic alcohol used in the pharmaceutical and food industry. It is used to protect food products against microorganisms during storage, as a flavoring in the production of chocolate and confectionery products, as an important ingredient in fragrance, and as a preservative in medical products. However, little is known of its effect on insects. The main aim of this study was to determine the influence of benzyl alcohol on the defense systems of the wax moth *Galleria mellonella*, i.e., its cuticular lipid composition and critical elements of its immune system. A gas chromatography/mass spectrometry (GC/MS) analysis found benzyl alcohol treatment to elicit significant quantitative and qualitative differences in cuticular free fatty acid (FFA) profiles. Our findings indicate that benzyl alcohol treatment increased the levels of HSP70 and HSP90 and decreased those of HSF1, histamine, and cysteinyl leukotriene. Benzyl alcohol application also increased dismutase level in the hemolymph and lowered those of catalase and 8-OHdG. The treatment also had negative effects on *G. mellonella* hemocytes and a Sf9 cell line in vitro: 48-h treatment resulted in morphological changes, with the remaining cells being clearly spindle-shaped with numerous granules. The high insecticidal activity of compound and its lack of toxicity towards vertebrates suggest it could be an effective insecticide.

## 1. Introduction

The remarkable evolutionary success of insects is partly due to their ability to build up a sophisticated, effective, and highly adaptable defense system against numerous microorganisms, including pathogenic fungi. Insects possess a complex and efficient system of defense against pathogens and parasites. The first line of defense against biological pathogens or insecticides is a cuticle formed of several layers, with an epicuticle on the outside, a procuticle underneath, and an epidermis beneath that. The epicuticular layer is also covered by wax, which plays an important role as a barrier against water [1,2,3]. About 70% of the cuticle is typically composed of proteins, with the remaining 30% being made up of chitin, lipids, chinons, and phenols. The insect cuticle is also covered with complex mixtures of mainly nonpolar and polar compounds [2,3,4].

The lipids of the cuticle may contain a range of hydrocarbons, free fatty acids (FFAs), alcohols, waxes, glycerides, aldehydes, and sterols. It is possible that specific cuticular lipids play a role in prey recognition by specialized and predatory insects. Indeed, the FFA profile can differ between insect groups and, sometimes, between the developmental stages of the same species; it has also been proposed that some cuticular lipids may have a role in defending against fungal invasion [2,3,5,6].

Insects rely on multiple immune responses that include both humoral and cellular defense reactions. Humoral reactions include the production of antimicrobial peptides (AMPs), reactive oxygen and nitrogen species, and the prophenoloxidase (proPO)-activating system, which regulates the coagulation and melanization of hemolymph. Cellular responses include the phagocytosis of small pathogens such as bacteria and fungi and the encapsulation of parasites such as parasitoids and nematodes, or nodulation by specific immune cells (hemocytes) [7,8]. It has been suggested that five classes of hemocytes are present in the Lepidoptera: prohemocytes, plasmatocytes, granulocytes, spherulocytes, and oenocytes. In contrast, the *Drosophila* have been found to present prohemocytes, plasmatocytes, lamelloctes, and crystal cells [9,10,11].

**Figure 1:**
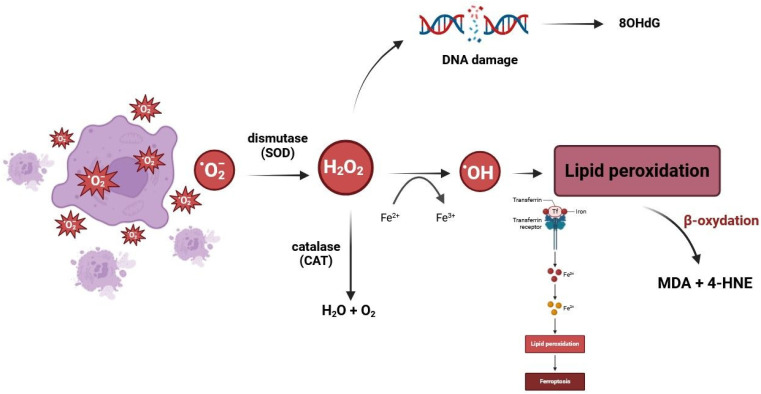
A general scheme of the interaction between reactive oxygen species (ROS) and the antioxidant defense system. The main sources of ROS generation include the mitochondrial electron transport chain, endoplasmic reticulum system, and NAD(P)H oxidase (NOX) complex. The oxygen utilized for respiratory purposes can be converted to ROS such as O^2−^, H_2_O_2_, and ·OH. Symbols used: H_2_O_2_—hydrogen peroxide; SOD—superoxide dismutase, CAT—catalase; MDA—malondialdehyde; 4-HNE—4-hydroxynonenal; 8OHdG—8-hydroxy-2′-deoxyguanosine.

Reactive oxygen species (ROS), like singlet oxygen, ·OH radical, or H_2_O_2_, can play a dual role. While they perform many important functions, such as regulating repair processes, metabolism, and gene expression, they also have very harmful effects [12]. In eukaryotic cells, ROS are mainly produced by the mitochondria. Under normal physiological conditions, approximately 95% of oxygen entering the mitochondrial electron transport chain is reduced to water molecules in the presence of cytochrome oxidase. The remaining 5% give rise to oxygen radicals, which are effectively removed by numerous defense mechanisms. Exceeding certain levels of ROS in the cell exacerbates oxidative stress, which may lead to the development of many free-radical diseases [13,14]. However, various enzymatic and non-enzymatic mechanisms form an effective control system to counteract the effects of increased free-radical production (Figure 1).

One of the most frequently studied processes related to the production of ROS and their effects is lipid peroxidation, i.e., the free-radical oxidation of unsaturated fatty acids resulting in the formation of peroxides [12,13]. The lipid peroxidation process consists of three main stages: initiation, propagation, and termination. This process is an avalanche and free radical in nature. The peroxidation reactions are particularly intense in cells exposed to oxidative stress, e.g., during infections, inflammations, neurodegenerative diseases, or cancer. It can result in damage to the cytoplasmic and mitochondrial membranes and their depolarization, and thus increased production of harmful free radicals in the cell.

Oxidative stress is a state of imbalance between oxidation and antioxidation. It is characterized by increased production of ROS, which destabilize their scavenging systems. High concentrations of ROS result in oxidative damage to proteins, lipids, and nucleic acids, disrupting homeostasis and ultimately leading to cell death [15,16]. However, organisms have developed effective antioxidative defense systems. The presence of antioxidant systems has also been found in insects [15,17,18,19,20], the main components of which include superoxide dismutase (SOD), catalase (CAT), peroxidase (POD), glutathione-S-transferases (GSTs), polyphenol oxidase (PPO), and ascorbate peroxidase (APX).

It is known that infections with entomopathogenic fungi influence processes related to oxidative stress. Chaurasia and colleagues [21] showed that the level of MDA, i.e., malondialdehyde derived from lipid peroxidation products in the small intestine and the fat body of *Periplaneta americana* increases after exposure to the fungi *Metarhizium anisoploae*, *Isaria fumosoroseus*, and *Hirsutella thompsonaii*; in addition, the level of MDA in the hemolymph was decreased. Tu and his team examined the effect of gamma-aminobutyric acid (GABA) and its potential mechanism on silkworms (*Bombyx mori*). GABA significantly increased the glutathione (GSH) content and activities of dismutase (SOD) and catalase (CAT) and reduced the level of MDA [22]. Also, Meng and colleagues [23] examined the effects of UV light irradiation on total antioxidant capacity, MDA and protein carbonyl content, and the activities of SOD, CAT, peroxidases (POXs), and glutathione-S-transferase (GST) in *Helicoverpa armigera* adults. They found that exposure to UV light for 30 min resulted in increased total antioxidant capacity, protein carbonyl content, and SOD, CAT, POX, and GST activities [23]. Also, Dong worked on the effects of ultraviolet light stress on protective and detoxification enzymes in insects [24]. Moreover, it has been reported that temperature stress, including high and low temperatures, could alter the balance between the generation of ROS and antioxidant defenses [25]. Li found that exposure of *Neoseiulus barkeri* to 40 °C resulted in the upregulation of the three protective enzymes SOD, CAT, and POD, with their levels first increasing, then decreasing, and finally increasing again [26].

Other important players in cell signaling and immunity are heat shock proteins (HSPs). Many studies suggest that HSPs activate immunity by binding to receptors on the cell surface [27,28,29]. Moreover, it has been suggested that HSPs constitute a link between innate and adaptive immunity, in particular HSP90, HSP70, and HSP60 are the most important players in the functioning of the mammalian immune system. Wrońska in her previous studies on *Galleria mellonella* larvae proved the presence of four HSPs (HSP90, HSP70, HSP60, and HSP27) [30], while other researchers identified heat shock proteins in insects from the orders Lepidoptera, Diptera, Hymenoptera, Psocoptera, and Hemiptera [31]. Wrońska’s results also confirm that *C. coronatus* infection increases the level of HSP27, HSP60, and HSP90 in wax moths. Wojda et al. indicated that injection of entomopathogenic *Bacillus thuringiensis* cells causes the HSP90 level in the fat bodies of *G. mellonella* larvae to increase approximately twofold 1.5 h after infection [32].

The main aim of the present work is to examine the effect of benzyl alcohol on the two major defense systems of insects: cuticular free fatty acid (FFA) profiles and hemocytes, i.e., part of the immune system. The testable hypothesis was that exposure of *G. mellonella* larvae to benzyl alcohol affects the presence of heat shock factor 1 (HSF1), cysteinyl leukotriene, and heat shock proteins (HSP-27, 60, 70, 90), which are essential elements of the immune system in mammals. It also measures certain elements of the oxidative chain reaction (catalase, dismutase, and DNA damage processes). Our research assumes that food additives may also act as effective insecticides; they offer the added advantage that they have been confirmed as safe for human consumption by the World Health Organization (WHO). However, despite their potential and growing interest from researchers, the use of food additives as insecticides and their effects remain poorly understood [33,34,35,36,37].

## 2. Results

### 2.1. The Effect of Benzyl Alcohol Application on Insects

Our findings showed that benzyl alcohol applied topically had no effect on the development or metamorphosis of *G. mellonela*. The most sensitive were the adults: mortality was 93 ± 6% after 48 h (Table 1) compared to 11 ± 3% in larvae.

**Figure 2:**
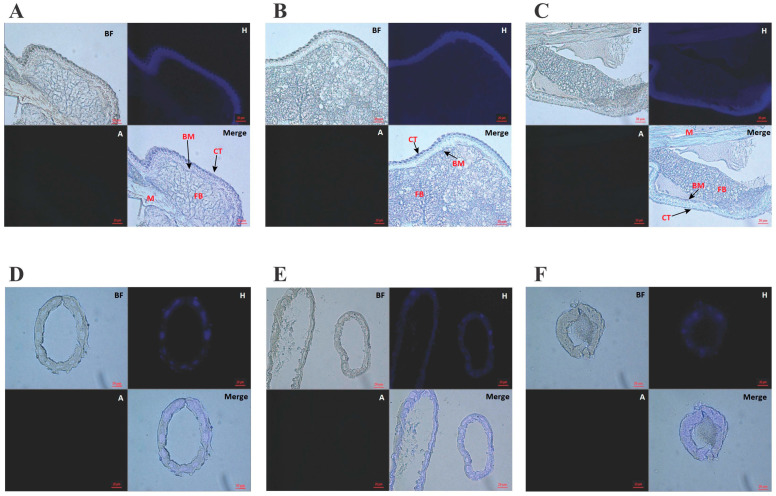
Histological sections of *G. mellonella* larvae stained with the Click-iT^®^ Plus TUNEL Assay for in situ Apoptosis Detection kit. (**A**–**C**): sections of *G. mellonella* larvae (middle body part; scale bar 20 μm); (**D**–**F**): sections of Malpighian tubules of *G. mellonella* larvae (scale bar 20 μm); (**A**,**D**): control insects; (**B**,**E**): acetone control (insects treated with acetone); (**C**,**F**): insects 24 h after administration of benzyl alcohol. Symbols: BF—brightfield; H—Hoechst 33342 (excitation 352, emission 455); A—Fluor 488 (excitation 493, emission 520); CT—cuticle; FB—fat body; BM—basement membrane; M—muscles.

The topical application of benzyl alcohol did not have any effect on the morphology of the treated insects. In addition, benzyl alcohol treatment did not have any effect on the morphology of the larval cuticle, basement membrane, muscles, fat body, Malpighian tubules, or any other studied vital organs and tissues, as indicated by histology examination, nor did it cause any apoptotic changes in the main body structures, as indicated by TUNEL staining (Figure 2).

Larvae were selected for the experiment, as they are more resistant to benzyl alcohol than the adults, thus allowing for of the effect of the compound on the activation of defense processes to be studied more. The latter are more likely to present decay and cell death, which would obscure the effects of the agent; they are also more difficult to collect hemolymph from. Furthermore, the immune system of *G. mellonella* larvae is far better understood than that of the adult moths, although this would be worth investigating in the future.

### 2.2. The Cuticular FFA Profiles of Galleria mellonella after Benzyl Alcohol Treatment

The GC/MS analysis indicated both quantitative and qualitative differences in cuticle FFA content between control *G. mellonella* and those treated with benzyl alcohol (Table 2). The total cuticular FFA content in control larvae was 485.49 µg/g of body mass, and 15 compounds from C4:0 to C20:1 were identified; of these, the highest concentrations were observed for C14:0 (28.69 µg/g), C16:0 (329.53 µg/g), and C18:1 (80.19 µg/g). The control adults demonstrated significantly higher total cuticular FFA content (6417.28 µg/g of body mass), and 16 FFAs were found from C6:0 to 20:1. The concentrations of the individual FFAs extracted from larvae and adults are presented in Table 2. It should be noticed that the FFAs identified in both developmental stages were present at higher concentrations in adults than in larvae. The cuticular FFA profiles of untreated larvae and adults differed significantly: larvae lacked C11:0, C12:0, C13:0, and C18:2, which are present in adults. In turn, C4:0, C5:0, and C7:0 are present in larvae but disappear after metamorphosis. Significantly higher amounts of C8:0, C9:0, C16:1, C18:1, and C18:0 were measured in control adults compared with control larvae (Table 2).

In the larvae, the highest total FFA content was observed after acetone treatment (934.79 μg/g of insect body); this value was 1.92 times higher than in the untreated control (485.49 μg/g of insect body) and only 1.07 times higher than in benzyl alcohol group (868.61 μg/g of insect body). On the other hand, in adults, the highest cuticular total FFA content was observed in the control (6417.28 μg/g of insect body); the value was 3.16 times higher than in the extract from insects treated with acetone (2027.21 μg/g of insect body), 11.86 times higher than in the benzyl alcohol group (541.17 μg/g of insect body), and 13.21 times higher than in the control larvae.

Treatment of *G. mellonella* larvae with benzyl alcohol resulted in a 1.78-fold increase in total cuticular FFA content. Furthermore, benzyl alcohol application resulted in the loss of C4:0, C5:0, C7:0, C10:0, C14:1, and C20:1; however, it should be noted that C4:0, C14:1, and C20:1 also disappeared after the application of solvent (acetone) alone, so only the loss of C5:0, C7:0, and C10:0 can be treated as a true effect of benzyl alcohol. In adults, treatment with benzyl alcohol resulted in the loss of C6:0, C8:0, C10:0, C12:0, C13:0, C15:0, and C18:2, as well as in a significant decrease in C11:0, C14:0, C16:1, C16:0, C18:1, and C18:0 compared with both controls; in all these cases, the reduction was partly due to acetone. However, the C20:1 level slightly increased after benzyl alcohol treatment, and a new C17:0 FFA was observed (Table 2).

Topical application of benzyl alcohol to the cuticle of adult moths had a much more serious effect on the FFAs profile than the same treatment of larvae. While benzyl alcohol caused the disappearance of three FFAs in larvae (C5:0, C7:0, C10:0), seven FFAs disappeared from the cuticle of adult insects (C6:0, C8:0, C10:0, C12:0, C13:0, C15:0, C18:2) and a new FFA, C17:0, appeared, which was absent in both control adults and control larvae. These changes were not caused by acetone.

### 2.3. The Effect of Benzyl Alcohol on Galleria mellonella Hemocytes and Sf9 Cells Cultured In Vitro

**Figure 3:**
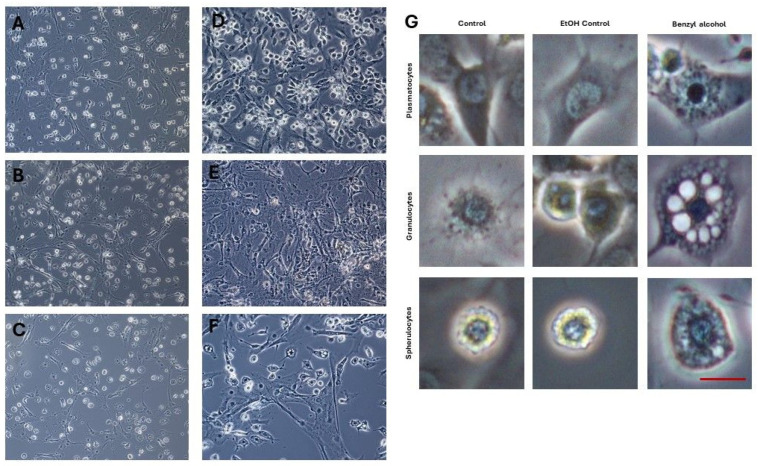
In vitro culture of hemocytes obtained from *G. mellonella* wax moth larvae. Control (**A**,**D**), ethanol control (1 μL 99.8% ethanol, final concentration 2.25 μg/μL) (**B**,**E**), and benzyl-alcohol-treated hemocytes (final concentration 0.28 μg/μL) (**C**–**F**). (**A**–**C**) 24 hpa—group 24 h after benzyl alcohol application, (**D**–**F**) 48 hpa—group 48 h after benzyl alcohol application. Scale bar 40 μm. (**G**)—Individual cell classes present in larvae hemocoel: control, ethanol control, and 48 h after benzyl alcohol application. Scale bar: 10 μm.

**Figure 4:**
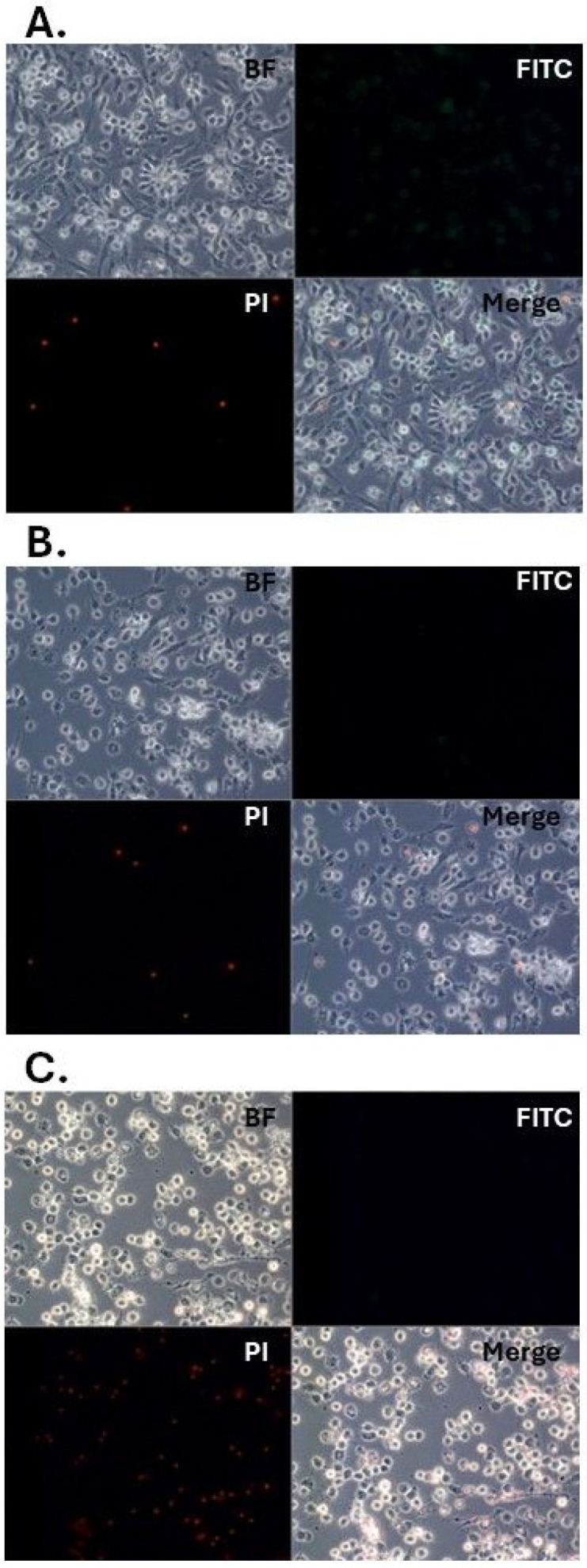
In vivo culture of *G. mellonella* larvae hemocytes: benzyl alcohol was topically administered. (**A**)—control insects; (**B**)—acetone control (insects treated with acetone); (**C**)—insects 24 h after administration of benzyl alcohol. PI—propidium iodide; FITC—fluorescein isothiocyanate.

**Figure 5:**
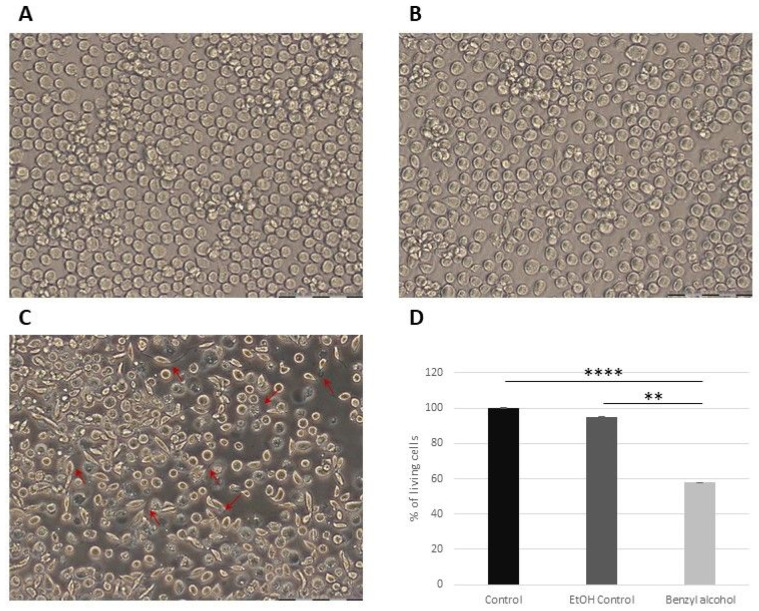
The morphological (**A**–**C**) and livability (**D**) changes in Sf9 cells after benzyl alcohol treatment: (**A**) Sf9 control group, (**B**) ethanol control group (1 µL 99.8% ethanol, final concentration 2.25 µg/µL), and (**C**) benzyl-alcohol-treated group (final concentration 0.28 µg/µL) cell lines. Red arrow—changes in cell shape and degranulation. Scale bar: 20 µm. (**D**) Graph showing percent of living Sf9 cells 24 h after administration of benzyl alcohol (final concentration 0.28 µg/µL), obtained using the WST-1 cell proliferation assay; data are expressed as mean ± SD; ** *p* < 0.01, **** *p* < 0.0001. Each test was performed in 3–8 independent replicates.

The effect of benzyl alcohol on the *G. mellonella* hemocytes and Sf9 cell morphology was documented at 24 and/or 48 h after application, i.e., topical application on the cuticle or direct application to cell cultures. Benzyl alcohol demonstrated an inhibitory effect with high toxicity to all tested types of cells (Figure 3, Figure 4 and Figure 5). In vitro and in vivo microscopic examination of *G. mellonella* hemolymph found that the treatment appeared to influence five cell types: prohemocytes, plasmatocytes, granulocytes, spherulocytes, and oenocytes. After 24 h of cultivation of the untreated hemocytes, prohemocytes and oenocytes disappeared, the spherulocytes were present as floating cells, and the plasmatocytes formed a characteristic network with the granulocytes. The tested compound appeared to have an inhibitory effect, with high toxicity against cells. Benzyl alcohol treatment (in vitro and in vivo experiments) resulted in changes in cell shape and disturbances in the production of networks between cells; 48 h after administration, the hemocyte cultures demonstrated inhibited network formation by plasmatocytes and granulocytes, together with cell disintegration (44.13 ± 13.88% of cells in the 48 hpa group); in addition, numerous large vacuoles were visible in the cells (47.17 ± 20.06% of cells in the 48 hpa group). No signs of apoptosis were observed in hemocytes collected from larvae treated with benzyl alcohol (Figure 4).

Sf9 cells treated with benzyl alcohol also demonstrated significant shape changes and cell degranulation compared to controls (Figure 5), with many spindled and disintegrated cells. Benzyl alcohol was found to be toxic against Sf9 cells, with less than 60% of the live cells observed 24 h after application (WST-1 test). The WST-1 test was not performed on the *G. mellonella* hemocytes as they appear unable to proliferate in vitro.

### 2.4. Quantitative Determination of Cysteinyl Leukotriene, Histamine, HSF1, and HSPs by ELISA Tests

**Figure 6:**
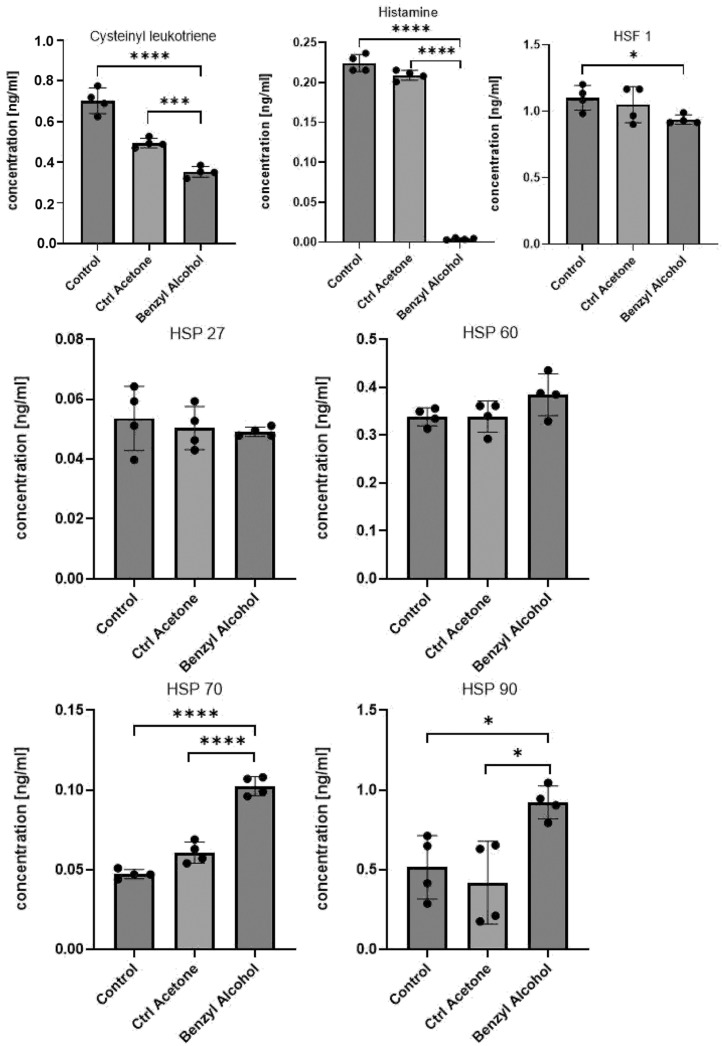
Changes in concentration of cysteinyl leukotriene, histamine, HSF1, HSP27, HSP60, HSP70, and HSP90 in *G. mellonella* hemolymph after benzyl alcohol administration, determined by ELISA (Enzo Life Sciences). Data are expressed as mean ± SD; * *p* < 0.05, *** *p* < 0.001, **** *p* < 0.0001. Each test was performed in four independent replicates (hemolymph collected from 8 to 10 larvae in each replicate).

The effect of benzyl alcohol treatment on the concentrations of cysteinyl leukotriene, histamine, HSF1, and four HSPs (90, 70, 60, and 27) in full hemolymph (lysed cells and plasma) of *G. mellonella* was also tested. The data were collected by ELISA, and the results were presented as means with SD (standard deviation) Figure 6). In all cases, the samples demonstrated equal variances (Brown–Forsythe test), and the population was normally distributed (Kolmogorov–Smirnov test) (*p*  < 0.05).

The treatment reduced cysteinyl leukotriene (Figure 6) levels: 0.70  ±  0.06 ng/mL in controls, 0.49  ±  0.02 ng/mL in the acetone group, and 0.35  ±  0.03 ng/mL in the benzyl alcohol group. Statistically significant differences were found between all groups: Contrast 1 (Control vs. benzyl alcohol): df  =  6, *p*  <  0.0001; Contrast 2 (Control vs. Ctrl acetone): df  =  6, *p*  =  0.0008; Contrast 3 (Ctrl acetone vs. benzyl alcohol): df = 6, *p* = 0.0002.

Treatment also decreased histamine (Figure 6) levels in *G. mellonella* larvae hemolymph relative to controls. The values were 0.22  ±  0.01 ng/mL in the control group, 0.21  ±  0.01 ng/mL in the control acetone group, and 0.004  ±  0.001 ng/mL in the benzyl alcohol group. The differences between groups were statistically significant. Contrast 1 (Control vs. benzyl alcohol): df  =  6, *p*  <  0.0001; Contrast 2 (Control vs. Ctrl acetone): df  =  6, *p*  <  0.04, Contrast 3 (Ctrl acetone vs. benzyl alcohol) df  =  6, *p*  <  0.0001.

The HSF1 level was slightly lower after benzyl alcohol application (Figure 6). Its concentration was 1.10  ±  0.09 ng/mL in the control group, 1.05  ±  0.13 ng/mL in the acetone control group, and 0.93  ±  0.03 ng/mL in the benzyl alcohol group. Statistically significant differences were observed only between the control and benzyl alcohol groups, Contrast 1 (Control vs. benzyl alcohol): df  =  6, *p*  =  0.01); therefore, it cannot be ruled out that the observed effect is the result of acetone administration.

Quantitative ELISA tests showed 0.52 ± 0.03 ng/mL of HSP 90 in control, 0.42 ± 0.04 ng/mL in ctrl acetone, and 0.92 ± 0.02 ng/mL after benzyl alcohol application. These results are statistically significant: Contrast 1 (Control vs. benzyl alcohol): df  =  6, *p*  <  0.01; Contrast 2 (Ctrl acetone vs. benzyl alcohol): df = 6, *p* = 0.01. HSP70 was present at 0.047  ±  0.002 ng/mL in the control group, 0.060  ±  0.006 ng/mL in the control acetone group, and 0.100  ±  0.005 ng/mL after benzyl alcohol treatment. All results are statistically significant: Contrast 1 (Control vs. benzyl alcohol): df  =  6, *p* < 0.0001; Contrast 2 (Control vs. Ctrl acetone): df  =  6, *p*  =  0.009; Contrast 3 (Ctrl acetone vs. benzyl alcohol): df = 6, *p* < 0.0001. HSP60 was also detected in all groups, but differences are not statistically significant. The concentration of HSP60 in the control group was 0.35  ±  0.02 ng/mL, 0.34  ±  0.04 ng/mL in the acetone group, and 0.38  ±  0.05 ng/mL in the benzyl alcohol group. Finally, HSP27 demonstrated a slight decrease after benzyl alcohol application, but the results are not statistically significant: the concentrations were 0.053  ±  0.004 ng/mL in the control group, 0.050  ±  0.011 ng/mL in the acetone group, and 0.049  ±  0.006 ng/mL in the benzyl alcohol group. All results for HSPs (90, 70, 60, and 27) are presented in Figure 6.

### 2.5. Benzyl-Alcohol-Activated Oxidative Defense Processes in Galleria mellonella

**Figure 7:**
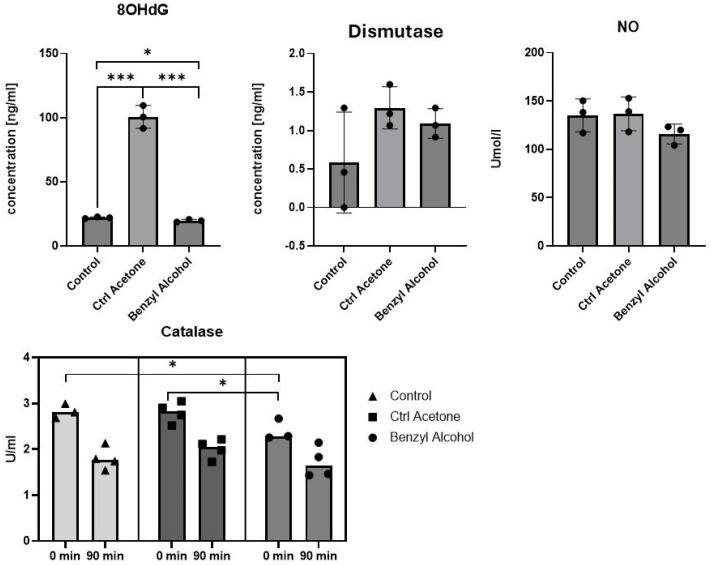
Changes in concentration of 8-OHdG, dismutase, NO, and catalase activity in *G. mellonella* hemolymph after benzyl alcohol administration, determined by ELISA (Enzo Life Sciences). Data are expressed as mean ± SD; * *p* < 0.05; *** *p* < 0.001. Each test was performed in three independent replicates (hemolymph collected from 8 to 10 larvae in each replicate).

This study also examined the effects of benzyl alcohol on 8-OHdG, dismutase, NO, and catalase (Figure 7) activities in wax moth hemolymph.

Significant differences in the 8-OHdG concentration were observed between untreated larvae (22.04 ± 0.65 ng/mL) and those treated with acetone (100.57 ± 8.91 ng/mL; *p* = 0.0001) and benzyl alcohol 19.70 ± 1.00 (*p* = 0.027). Interestingly, acetone alone caused an increase in 8-OHdG concentration, while benzyl alcohol administered with a carrier (acetone) caused the opposite effect, i.e., a decrease in concentration. The dismutase activity was lower in the control group (0.58 ± 0.53 ng/mL) than the benzyl alcohol (1.09 ± 0.15 ng/mL) and acetone group (1.29 ± 0.22 ng/mL), but not significantly. A small decrease in the NO level was observed after benzyl alcohol application, but this was not significant. The catalase level was 2.83 ± 0.13 U/mL in the control group, i.e., significantly higher (both 0 and 90 min) than after benzyl alcohol administration (2.40 ± 1.71 U/mL). Catalase level at time point 0 after acetone treatment was at the same level as in the untreated control and decreased after 90 min.

## 3. Discussion

Benzyl alcohol is a transparent liquid with a faint aromatic odor and a sharp burning taste. It is generally recognized as safe for consumption (CASRN: 100-51-6; molecular weight of 108.13) (E 1519) and is authorized for use as a food additive in the EU in accordance with Annex III to Regulation (EC) No 1333/2008. It is also an ingredient (5%) in Ulesfia Lotion (Sciele), approved by the U.S. Food and Drug Administration (FDA) for the treatment of head lice in patients aged six months or over [38]. A study of 695 subjects in all phases of clinical development found it to be a safe and effective, non-neurotoxic topical head louse treatment in this age group (*p* < 0.001). The scanning electron micrograph (SEM) analysis found the active substance to asphyxiate the lice by stunning/plugging the open breathing spiracles [38]. Benzyl alcohol is also used to preserve pharmaceutical substances during storage (as a 2% additive), as a flavoring in chocolate and confectionery products, wines, and aromatized liqueurs, and as a component of fragrance [39,40]. Furthermore, benzyl alcohol is also present in nature and is naturally produced by many plants and can be detected in extracts of tea leaves, ylang-ylang, jasmine, and hyacinth, also comprising one of the major floral volatile compounds in *Prunus serotina* and other *Prunus* species [41,42].

It is important that the tested substances can penetrate the cuticle, as this is the standard mode of entry for insecticides. The work of Webb and Green [43] shows that benzyl alcohol administered topically to *Melophagus ovinus* causes immobilization of insects after 2 h and death after 21 h, suggesting penetration of this compound through their cuticle. The passage of benzyl alcohol from the outer surface of the insect into the subcutaneous tissue may occur via lipophilic elements, along which the wax secretions of the hypodermal cells pass to the epicuticle, and the pore canals, passing from the subcutaneous tissue to the base of the epicuticle, may facilitate diffusion [43]. Penetration of benzyl alcohol through artificial beeswax membranes lasted 32 min, indicating a relatively low efficiency of this process [43]; therefore, in our studies, benzyl alcohol was applied to *G. mellonella* larvae and adults together, with acetone used as a solvent. Acetone is widely used for administration of chemical substances and bio-active compounds to insects [44]. Preliminary (unpublished) data have shown the presence of benzyl alcohol in the hemolymph of *G. mellonella* larvae topically treated with this substance. However, the efficiency of benzyl alcohol penetration through the cuticle of *G. mellonella* remains unknown, and it is unclear whether such penetration differs between larvae and adults due to the fundamentally different structures of their cuticles [45]. Further studies are necessary to verify whether the efficiency of benzyl alcohol penetration through the cuticle is the same in all developmental stages of *G. mellonella*.

In our experiments, insects treated with benzyl alcohol did not demonstrate any morphological changes in their internal organs, e.g., the Malpighian tubules involved in excretion and analogous to kidneys [46,47]. This might suggest either a rapid metabolism or good tolerance. Furthermore, it is also unclear whether the benzyl alcohol, after being applied to the surface of the insect body and passing through the cuticle, is passively or actively transported inside the host, and its methods of distribution and metabolism remain unknown.

The choice of solvent is also a key consideration in the topical application procedure. In all experiments with *G. mellonella*, acetone was used as a solvent. Acetone has been found to possess relatively low acute toxicity for mammals and aquatic and terrestrial arthropods and is frequently used in physiological and biochemical investigations on insects [44]. Acetone is carboxylated to acetoacetate by bacteria, and is incorporated into fatty acids during lipogenesis in mammal adipose tissue [48,49]. The above data should be considered when assessing the effects of benzyl alcohol administration on the cuticle lipid profile. However, no data currently exist on the metabolism of acetone in insects or the presence of any enzymatic machinery that can convert acetone to FFAs in insect tissues.

Our present findings indicate that topical application of benzyl alcohol had profound effects on the cuticular FFA profiles in *G. mellonella*, resulting in qualitative and quantitative changes which were dependent on the developmental stage. The FFA profiles of the control larvae and adults of *G. mellonella* were similar to those described previously [50,51,52]. Differences were observed between developmental stages, with 15 different saturated and unsaturated fatty acids, ranging from C6:0 to C20:1, being noted in untreated larvae and 16 from C4:0 to C20:1 in adult moths. FFAs C16:0 and C18:1 predominated in all developmental stages. In the *G. mellonella* larvae, benzyl alcohol treatment resulted in a 1.8-fold increase in total cuticular FFAs compared to untreated larvae. Considering that acetone alone caused a 1.9-fold increase, the effect of benzyl alcohol on the increase in total cuticular FFAs concentration can be excluded. In adults, the highest cuticular total FFA content was observed in the control; the value was 3.2 times higher than in insects treated with acetone and 11.9 times higher than benzyl alcohol.

Benzyl alcohol treatment resulted in the loss of C5:0, C7:0, and C10:0 from the larval cuticle, and acetone alone resulted in the loss of C4:0, C14:1, C18:0, and C20:1. It should be noted that C5:0, C7:0, and C10:0 were present in the control and acetone-treated larvae, so the disappearance of these FFAs can be attributed to the action of benzyl alcohol. In adults, benzyl alcohol treatment resulted in the loss of C6:0, C8:0, C10:0, C12:0, C13:0, C15:0, and C18:2 (all present in control and acetone-treated adults) and a significant decrease in C11:0, C14:0, C16:1, C16:0, C18:1, and C18:0 compared with both the control and acetone groups; acetone alone resulted in the disappearance of only C14:1. It cannot be excluded that the rise in total FFAs concentration observed following benzyl alcohol/acetone treatments of larvae might be associated with the destruction and release of FFAs from disintegrating hemocytes and/or other insect cells. It is also very likely that, depending on the stage of development, lipid metabolism is governed by completely different mechanisms. The fact that treatment with benzyl alcohol and acetone alone have different effects on the presence of individual FFAs in the cuticle of larvae and adults might suggest that cuticular lipids are subject to various metabolic pathways in both developmental stages. The disappearance of three FFAs (C5:0, C7:0, C10:0) from the cuticle of larvae and seven FFAs (C6:0, C8:0, C10:0, C12:0, C13:0, C15:0, C18:2) from the cuticle of adults is an obvious effect of benzyl alcohol application, because these FFAs were present in the cuticle of both acetone-treated and control insects. Also, the appearance of C17:0 in adults occurred only after benzyl alcohol application. Further studies are necessary to explain the basis for the different response of larvae and adults to benzyl alcohol administration.

Insects also employ a systemic response, i.e., the combined action of the cellular and humoral response, to defend themselves against pathogens [53,54,55,56,57,58]. The humoral immunity of insects is determined by low-molecular-weight immune peptides synthesized by the fat body and hemocytes. The cellular response involves hemocytes: specialized hemolymph cells which play a role in phagocytosis, encapsulation, nodulation, recognition of foreign bodies, and synthesis of peptides and immune proteins, as well as the initial stages of coagulation and melanization. In most arthropods, these two types of immune response work closely together and are activated immediately to kill the pathogen.

Our data indicate that benzyl alcohol has an effect on *G. mellonella* larvae hemocyte morphology after 24 and 48 h in vitro. While the control cultures all presented species-specific hemocyte types with properly formed cellular networks, benzyl alcohol application altered cytoskeleton organization and demonstrated toxic effects against both the Sf9 cell line and *G. mellonella* hemocytes. The treatment affected cell structure, with the cells becoming more spindled, and lacking the network characteristic of hemocytes. After 48 h, the cells were found to have disintegrated, and hemocyte classification was almost impossible. Sf9 cells (derived from the ovary of the *Spodoptera frugiperda* pupa) [59,60,61,62] for decades been a useful model to evaluate the potential of certain substances to act as insecticides as well as to investigate potential resistance mechanisms [62]. Here, we used Sf9 cells as a model to investigate the toxic effect of benzyl alcohol. The WST-1 test confirmed that benzyl alcohol effectively killed the Sf9 cells. However, benzyl alcohol was not found to take part in the stimulation of apoptosis.

Understanding the mechanisms of the interaction between the innate immune system and HSPs will make it possible to rationally modulate immune responses, either towards immunity or towards tolerance [30,63,64]. Wrońska and Boguś confirmed the occurrence of HSPs in *G. mellonella* larvae [30]. Our finding showed that levels of HSP70 and HSP90 increase after benzyl alcohol application. Both HSPs are highly abundant and ubiquitous molecular chaperones which play essential roles in many cellular processes, including cell cycle control and cell survival, and in hormone and other signaling pathways and are important factors in maintaining cellular homeostasis [63,64]. Following on from these findings, the present study examined also whether HSF1, histamine, and cysteinyl leukotriene are altered by the application of benzyl alcohol.

As in our present study, Sobich [65] also confirmed the presence of HSF1 and histamine, as well as cysteinyl leukotriene (CysLT) and Toll-like receptors (TLR 1 and TLR2) in *G. mellonella* larvae during *Conidiobolus coronatus* infection. CysLT level did not significantly differ between the control group and the infected larvae. Both histamine and HSF1 levels increased significantly after exposure to *C. coronatus* [65]. In contrast, our present findings indicate that all three, HSF1, histamine, and cysteinyl leukotriene, decreased after benzyl alcohol application. In the case of cysteinyl leukotriene and HSF-1, we cannot exclude the dominating effect of acetone. Histamine, a known insect neurotransmitter [66], was particularly sensitive to benzyl alcohol and was barely detectable 24 h after application. A study by Priyadarsini from 2019 [67] showed that infection of *Drosophila melanogaster* larvae with *Enterobacter ludwigii* bacteria had a major impact on histamine levels, and the infected fruit fly also started to release ROS into the hemolymph; this had a critical impact on the life cycle, appearance, and behavior of the insect. Interestingly, histamine was found to be absent in the infected flies [67].

Furthermore, antioxidant systems have also been found in insects [12,15,16,17,18,19,68,69]. Previous studies have found that infections with entomopathogenic fungi influence processes related to oxidative stress [12,70]. For example, the level of MDA, an indicator of oxidative stress, increases in *Periplaneta americana* insects after exposure to the fungi (*Metarhizium anisopliae, Isaria fumosoroseus,* and *Hirsutella thompsonii*) [70]. An increase in MDA was observed in the hemolymph and the entire body of this species. Increased glutathione peroxidase activity was also noted in the hemolymph, fat body, and midgut of *P. americana* exposed to *H. thompsonii* spores [21]. Similar results were reported by Hyrsl, where MDA activity was measured in the fat body and hemolymph of *G. mellonella* larvae exposed to boric acid [71]. A recent study in our group also confirmed the presence of compounds of the oxidative system in *G. mellonella* larvae hemolymph, and that their level was sensitive to benzyl alcohol application.

Insects have evolved a range of mechanisms based on enzymatic (dismutase, catalase, glutathione) and non-enzymatic antioxidant systems to mitigate the effects of oxidative damage. Under normal physiological conditions, each antioxidant enzyme interacts with the others. In the present study, benzyl alcohol application lowered the concentration of 8-OHdG and catalase activity (Figure 7). Lower levels of 8-OHdG, a DNA damage biomarker, may suggest that *G. mellonella* antioxidant enzymes can effectively counteract the prooxidant effect of benzyl alcohol on DNA damage. Significantly lower activity of catalase after benzyl alcohol application and no differences in the levels in dismutase or NO suggests that antioxidant enzymes are effective against the harmful effects of lipid peroxidation. Kazek [12], in her previous work, demonstrated that the level of glutathione peroxidase (GPx) was found to be elevated in the hemolymph after *C. coronatus* infection, while catalase and superoxide dismutase activities were lowered. Here, we did not measure the level of glutatione and its enzymes, which also play crucial role in the management of lipid peroxidation, and their dysfunction results in the accumulation of lipid peroxides and, potentially, cell death. Testing of two naturally occurring chemical compounds, benzyl alcohol and benzoyl benzoate, for insecticidal activity against *Tribolium castaneum* by Aboelhadid and co-workers [72] showed that benzyl alcohol had a significant insecticidal effect and inhibited acetylcholinesterase, decreased the level of reduced glutathione (GSH), and increased malondialdehyde (MDA) concentration in treated *T. castaneum*. Another group of researchers Abdel-Azeem et al. [73] obtained a similar effect after using benzyl alcohol against *Acanthoscelides obtectus*.

The effect of benzyl alcohol on animal cells is complex and unclear in many respects. On the one hand, benzyl alcohol protects against acute liver damage induced by paracetamol and reduces inflammasome activation in a Toll-like-receptor-4-dependent manner [74]; reduces the toxicity of Shiga toxin, diphtheria toxin, and ricin [75]; and inhibits cytotoxicity induced by *N*-methyl-D-asparagine [76], but at the same time, it induces reversible fragmentation of the Golgi apparatus [75] and inhibits the activity of glycosyltransferases in the rat liver Golgi membrane [77]. The presence of benzyl alcohol in popular e-liquids caused a decrease in mitochondrial and cellular membrane potentials and an increase in ROS production in the human HEK2 cell line [78]. This flavoring agent suppresses the natural killer (NK) cell activity [79], abolishes the protective effect of superoxide dismutase in hepatocytes [80], and causes a variety of morphological defects in developing zebrafish embryos [81].

Benzyl alcohol is known as a membrane “fluidizer” that affects lipid bilayer structure [82] as well as membranes of erythrocytes and hepatocytes [83,84]. It seems possible that this compound affects the cell membrane of insect cells in a similar way, causing changes in the shape of *G. mellonella* hemocytes and Sf9 cells.

To summarize, we present our results concerning the effect of benzyl alcohol application on the defense systems of *G. mellonella*, i.e., its cuticular lipid composition and critical elements of the immune system. Our findings demonstrate significant quantitative and qualitative changes in cuticular FFA profiles of benzyl-alcohol-treated insects, the cellular and humoral immune response in *G. mellonella* larvae, and the connection between this process and those associated with oxidative stress. In most cases, we were able to distinguish the effects of benzyl alcohol from those of its carrier, acetone. However, further studies using other carriers are necessary to gain a more comprehensive insight into the effects of benzyl alcohol on essential components and mechanisms ensuring homeostasis in *G. mellonella.* None the less, considering that the compound is safe enough to be authorized as a food additive in the EU, benzyl alcohol offers considerable potential as an insecticide against both lice and the bee pest *G. mellonella.* Recent reports also mention the possibility of using benzyl alcohol against red flour beetle *T. castaneum* and dried bean beetle *A. obtectus* [72,73].

## 4. Materials and Methods

### 4.1. Chemical Reagents

The benzyl alcohol (>99%, liquid) was obtained from Sigma Aldrich (product number 108006; CAS Number 100-51-6, St. Louis, MO, USA). The compound was dissolved in acetone (Avantor, Gliwice, Poland) or ethanol (Avantor, Gliwice, Poland) and given to both larvae and adults of *G. mellonella* topically (in vivo condition) or directly to hemocyte or Sf-9 cell culture (in vitro condition). It is worth emphasizing that ethanol was used as the solvent for benzyl alcohol in the in vitro experiments, as acetone is toxic for cells and dissolves polystyrene plates. However, acetone was used as the solvent for topical application of benzyl alcohol, as it effectively penetrates the cuticle, while ethanol does not.

### 4.2. Insects

All insects used in experiments were reared in the laboratory under optimal growth conditions. The wax moth *G. mellonella* was reared in glass chambers at 30 °C, 70% relative humidity, and in constant darkness. The insects were maintained on a semi-artificial diet, as described by Sehnal [85]. Five-day-old last instar larvae (wandering stage), which had ceased feeding, and mature six-day-old adults were used for the experiments. Adults were used to check the toxic effect of benzyl alcohol and measure the mortality after administration. Both larvae and adults were used in studies of cuticular free fatty acid (FFAs) profiles.

### 4.3. Benzyl Alcohol Application for Experiments

For the in vivo experiments, benzyl alcohol was directly administered to the dorsal side of the wax moth cuticle by topical application. During the experiments, the substance was dissolved in 99.8% acetone, allowing for good penetration of the cuticle [44]. Each insect was treated with 5 μL of the mixture containing 100 μg of benzyl alcohol. The dose employed was approximately LD70 and LD90 after 24 h and 48 h application, respectively. After benzyl alcohol application, the insects were reared in optimal growth conditions, as described above. The insects were collected 24 h after treatment and kept at −80 °C until analysis.

On the other hand, during the in vitro experiments, 1 μL of ethanol containing 100 μg of benzyl alcohol was added to cell suspension (SF9 cell line or hemocytes of wax moth larvae), giving a final concentration of 0.28 μg/μL. For the control cultures, 1 μL volumes of 99.8% ethanol were added to the cell cultures, and the cell culture plates (Nest, Wuxi, China) were incubated for another 24 or 48 h, depending on the group, at 30 °C and 80–90% humidity.

### 4.4. Mortality Observations

After topical administration of benzyl alcohol (upper dorsal part of insect body) on both wax moth adults and larvae, insects were put to new fresh glass jars (moths) and Petri dishes (larvae). Then, they were reared in the laboratory under optimal growth conditions. The insect mortality rate was checked in 1, 24, and 48 h after application of the tested compound (3 independent replicates, each n = 15).

### 4.5. Sf9 Line and WST-1 Test

The Sf9 cell line (Thermo Fisher Scientific, Waltham, MA, USA) was cultured in optimal growth conditions at 27 °C in the appropriate TNM-FH medium (Merck, Darmstadt, Germany). The culture was passaged every five to seven days according to the manufacturer’s recommendations.

The experiments were conducted in 48-well culture plates (Nest, Wuxi, China), each well containing 300 µL of medium and 50 µL of stock, with approximately 2.5–3 × 10^5^ cells. The cells were cultured for 24 h, and then, 1 µL of ethanol containing 100 µg of benzyl alcohol (final concentration 0.28 µg/µL) was added as described previously [86]. The cells were then incubated for another 24 or 48 h, and changes in their morphology and activity were observed. Additionally, cell proliferation and viability were measured using the WST-1 test. Briefly, 30 µL of the WST-1 reagent (Roche, Basel, Switzerland) was added to each plate well, the absorbance was read at 440 and 650 nm after three hours, and the results were calculated according to the manufacturer’s instructions. Each test was carried out in three independent replicates. Only the Sf9 cell line was tested after benzyl alcohol treatment (*G. melonella* hemocytes do not proliferate in vitro).

### 4.6. Hemolymph Collection and Wax Moth Hemocyte Cultures

The hemocyte cultures were established from freshly collected hemolymph of *G. mellonella* larvae (collection hemolymph from adults its barely impossible). The larvae were first washed with distilled water and then immersed briefly in 70% (*v*/*v*) ethanol to sterilize their surfaces and reduce contamination. *G. mellonella* larval hemolymph was collected from small incisions in the last proleg (one drop of hemolymph 26 μL contained 1.3 × 10^5^ cells). Fresh hemolymph was collected into sterile polypropylene 1.5 mL centrifuge tubes preloaded with 300 μL Grace Insect Medium (GIM; Gibco, Billings, MT, USA) with added gentamycin (10 mg/mL; Gibco, Billings, MT, USA), amphotericin B (250 μg/mL; Gibco, Billings, MT, USA), and 0.1 mM phenylthiourea (PTU; Merck, Darmstadt, Germany). Subsequently, the hemolymph suspension was immediately transferred to 48-well culture plates with a glass bottom (Nest, Wuxi, China). Each plate well was filled with 100 μL of the hemocyte suspension (8–10 drops from 2 to 3 larvae were mixed with 300 μL of GIM) and supplemented to 400 μL with sterile GIM; this was then incubated for 24 h at 30 °C and 80–90% humidity. Following this, 1 μL of ethanol (Avantor, Gliwice, Poland) containing 100 μg of benzyl alcohol (Merck, Darmstadt, Germany) was added to the cell suspension, giving a final concentration of 0.28 μg/μL. For the control cultures, 1 μL volumes of 99.8% ethanol were added to the cell cultures and untreated cells, and the plates were incubated for another 24 or 48 h, depending on the group, at 30 °C and 80–90% humidity. Each hemocyte culture was performed as five or six independent replicates. The obtained hemocyte cultures were analyzed using an inverted AxioVert A1 fluorescence microscope with phase contrast, equipped with an Axio Cam ICc 5 camera and Zen lite 2012 software (Zeiss, Jena, Germany) and/or an Olympus (type IX 50) inverted phase contrast microscope with Color View camera (IIIu) connected with Cell D software 5.1 (Olympus, Tokyo, Japan).

For the in vivo experiments with benzyl alcohol, two control groups were used: one group that was not treated with anything, and a second group that was given 5 μL of acetone. Twenty-four hours after administration of the benzyl alcohol (100 μg in 5 μL acetone), hemocytes were collected from these larvae and cultured as described above. Necrosis and apoptosis were determined with the Annexin V-FITC apoptosis detection kit (Enzo Life Sciences, Farmingdale, NY, USA) using propidium iodide (PI) in the treated-insect hemocytes, where Annexin V is conjugated to FITC fluorochrome.

### 4.7. Immunohistochemical Visualization of Apoptosis

The effect of benzyl alcohol on the internal organs of *G. mellonella* was determined 24 h after topical application using last instar larvae (wandering stage). Untreated larvae and insects treated with acetone were used as controls. Firstly, the larvae were washed with distilled water, then immersed briefly in 70% (*v*/*v*) ethanol to sterilize their surfaces. The larvae were immediately fixed overnight at 4 °C in a solution of 4% paraformaldehyde (Merck, Darmstadt, Germany) in 0.1 M phosphate-buffered saline (PBS; Merck, Darmstadt, Germany), pH 7.4. After fixation, the larvae were washed three times in 0.1 M PBS and dehydrated in an increasing series of ethanol and chloroform (Chempur, Piekary Śląskie, Poland), then embedded in paraffin wax (melting temperature 53–57 °C; Merck, Darmstadt, Germany), cut into 8 μm-thick sections, and mounted on SuperFrost Plus glass slides (Thermo Fisher Scientific, Waltham, MA, USA). The sections were dewaxed in chloroform (10 min × 2), followed by consecutive 100%, 96%, and 80% ethanol (10 min each), and water, and then were stained to identify apoptotic cells using the Click-iT Plus TUNEL Assay for in situ Apoptosis Detection kit (Thermo Fisher Scientific, Waltham, MA, USA). Subsequently, the samples were again dehydrated in ethanol and xylene (Chempur, Piekary Śląskie, Poland) baths and mounted in HIGHDEF IHC fluoromount (Enzo Life Sciences, Farmingdale, NY, USA) for microscopy.

### 4.8. Enzyme-Linked Immunosorbent Assays

The next stage evaluated the oxidative stress processes taking place in the full hemolymph of the larvae and determined the levels of histamine, HSF1, Cysteinyl leukotriene, HSP27, HSP60, HSP70, and HSP90. Briefly, 15 larvae were placed on ice to anesthetize them, and the hemolymph was collected and mixed with 100 μL of PBS with 0.1 mM phenylothiourea (PTU; Merck, Darmstadt, Germany) to inhibit melanization. The processes associated with oxidative stress were measured 24 h after benzyl alcohol application using the following kits: Catalase Fluorometric Detection, Cu/Zn-Superoxide Dismutase ELISA, DNA Damage ELISA, and Glutathione Peroxidase Assay (all kits from Enzo Life Sciences, Farmingdale, NY, USA). The quantitative histamine, HSF1, and Cysteinyl leukotriene analyses were performed using commercial ELISA kits from Enzo Life Sciences. Quantitative HSP analyses were also carried out using ELISA tests from Enzo Life Sciences: HSP27 (human) ELISA kit, HSP60 (human) ELISA kit, HSP70 high sensitivity ELISA kit, and HSP90 (human) ELISA kit. Each test was performed in four independent replicates (n = 10 per one independent measurement) according to the manufacturer’s instructions. Absorbance was measured using a Synergy HTMicroplate Reader (BioTek, Winooski, VT, USA).

### 4.9. Extraction of Samples, Derivatization and GC/MS Analysis

All methods used for free fatty acids (FFAs) extraction and analysis were previously described in detail in references [5,6,50,51,86].

### 4.10. Statistical Analysis

Student’s *t*-test, Brown–Forsythe test, normality Kolmogorov–Smirnov test, and ANOVA were used to check the statistical significance of the results. A post hoc analysis was performed using the Tukey test. Results at the *p* ≤ 0.05 level were considered significant. The analysis was performed using the Prism 8.0 software (GraphPad Software, Boston, MA, USA) and Statistica 6.0 (StatSoft Polska, Kraków, Poland).

## 5. Conclusions

Insecticides are pesticides that are formulated to kill, harm, repel, or mitigate one or more species of insect. They have various modes of operation, for example, some can disrupt the nervous system while others may act as repellents, and they may find a range of applications from agricultural to household uses. Although each chemical insecticide typically has a single intended target, its use will result in a disruption of local biodiversity. We hypothesize that some selected food additives can play a significant role in controlling insect populations in nature and that they can be an integral part of integrated pest management programs in many ecological areas. It is necessary to understand the mechanisms of their actions. Unfortunately, little, if anything is known about the action of food additives on invertebrate species, especially in insects.

Our findings can have a considerable impact on pest control and improve the general understanding of the potential activity of food additives, i.e., as insecticides, in this regard. Such research into the basic mechanisms of their insecticidal properties can support the design of more effective and environmentally friendly means of controlling insect pests. Our findings serve as the first morphological and functional characterization of the action of food additives in various structures within Lepidopteran species.

## Figures and Tables

**Table 1:** Effect of benzyl alcohol on mortality of *G. mellonella* larvae and adults.

Time After Treatment (Hours)	Mortality (Mean % ± SD)	
	Larvae	Adults
Untreated:		
1	0	0
24	0	0
48	0	0
Acetone:		
1	0	0
24	0	0
48	11 ± 3	0
Benzyl alcohol:		
1	0	0
24	0	73 ± 13
48	11 ± 3	93 ± 6

Benzyl alcohol was applied as described in the Material and Methods Section (dose 100 µg; 3 independent replicates, each n = 15). The differential mortality of benzyl-alcohol-treated adults and larvae is statistically significant (*t*-test; *p* < 0.0001).

**Table 2:** Content of free fatty acids present on the cuticle of *Galleria mellonella* larvae and adults.

*Galleria mellonella* Larvae [µg/g of Body Mass ± SD]				*Galleria mellonella* Adults [µg/g of Body Mass ± SD]		
FFA	Control	Acetone	Benzyl Alcohol	Control	Acetone	Benzyl Alcohol
C4:0	0.72 ± 0.28	ND	ND	ND	ND	ND
C5:0	0.36 ± 0.13	0.28 ± 0.13	ND	ND	ND	ND
C6:0	1.11 ± 0.47	1.30 ± 0.37	0.77 ± 0.44	2.03 ± 1.04	0.63 ± 0.28	ND
C7:0	0.96 ± 0.34	1.17 ± 0.71	ND	ND	0.38 ± 0.33	ND
C8:0	1.63 ± 0.05 ^@^	2.28 ± 1.01	0.96 ± 0.61	4.36 ± 1.47 ^@A^	1.31 ± 0.61 ^A^	ND
C9:0	1.34 ± 0.17 ^#^	2.44 ± 0.75	4.02 ± 2.74	112.02 ± 6.48 ^#B^	56.97 ± 16.54 ^B^	1.25 ± 0.37 ^B^
C10:0	0.96 ± 0.30	0.68 ± 0.3	ND	4.38 ± 3.18	0.64 ± 0.32	ND
C11:0	ND	ND	ND	1268.38 ± 361.77 ^C^	432.38 ± 126.11 ^C^	32.26 ± 4.88 ^C^
C12:0	ND	ND	ND	23.94 ± 5.77 ^D^	2.40 ± 1.14 ^D^	ND
C13:0	ND	ND	ND	7.92 ± 0.25 ^E^	3.58 ± 1.51 ^E^	ND
C14:1	2.02 ± 1.35	ND	ND	12.12 ± 6.61	ND	ND
C14:0	28.69 ± 9.39 ^a^	16.59 ± 2.45	7.39 ± 5.81 ^a^	43.94 ± 13.15 ^F^	6.83 ± 2.37 ^F^	1.21 ± 0.28 ^F^
C15:0	7.12 ± 5.79	9.26 ± 4.41	3.10 ± 2.29	17.93 ± 11.25	3.25 ± 1.57	ND
C16:1	18.34 ± 13.72 ^$^	1.05 ± 0.33	38.87 ± 30.47	264.61 ± 9.36 ^$G^	29.98 ± 12.87 ^G^	5.10 ± 0.49 ^G^
C16:0	329.52 ± 154.13 ^%b^	842.77 ± 38.43 ^b^	631.70 ± 418.62	3687.79 ± 323.09 ^%H^	1057.30 ± 120.19 ^H^	236.95 ± 42.65 ^H^
C17:0	ND	ND	ND	ND	ND	0.20 ± 0.12
C18:2	ND	ND	ND	62.52 ± 84.21	14.28 ± 5.46	ND
C18:1	80.19 ± 49.57 ^&^	57.25 ± 17.89	168.19 ± 142.79	730.16 ± 142.87 ^&I^	344.56 ± 30.01 ^I^	220.95 ± 31.25 ^I^
C18:0	12.27 ± 2.72 *	ND	16.42 ± 5.54	168.87 ± 10.79 *^JK^	65.36 ± 27.82 ^J^	32.82 ± 2.59 ^K^
C20:1	1.33 ± 0.37 ^^^	ND	ND	6.30 ± 2.83 ^^^	7.38 ± 2.15	10.43 ± 4.31
Total	485.49 ± 238.81	934.79 ± 66.78	868.61 ± 500.83	6417.27 ± 984.09	2027.20 ± 349.25	541.17 ± 86.94

Significant differences between untreated larvae and adults are marked with the same special characters (@, #, $, %, &, *, ^); in addition, significant differences between the treated groups (acetone or benzyl alcohol) and controls are marked with the same letters: small letters (a, b) for larvae and capital letters (A–K) for adults. (ANOVA, Tukey’s HSD test, and *t*-test, *p* < 0.05). SD—standard deviation, FFA—free fatty acid, ND—not detected.

## Data Availability

The data presented in this study are available on request from the corresponding author due to waiting for acceptance of data submitted to the RepOD open repository.
